# Extracranial Artery Stenosis Is Associated With Total MRI Burden of Cerebral Small Vessel Disease in Ischemic Stroke Patients of Suspected Small or Large Artery Origins

**DOI:** 10.3389/fneur.2019.00243

**Published:** 2019-03-21

**Authors:** Tao Lu, Jiahui Liang, Ninglin Wei, Liya Pan, Hong Yang, Baohui Weng, Jinsheng Zeng

**Affiliations:** ^1^Department of Neurology and Stroke Center, The Fourth Affiliated Hospital of Guangxi Medical University, Liuzhou, China; ^2^Department of Neurology and Stroke Center, The First Affiliated Hospital, Sun Yat-Sen University, Guangzhou, China

**Keywords:** carotid artery diseases, ischemic stroke, cerebral small vessel diseases, magnetic resonance imaging, total burden

## Abstract

**Background and Purpose:** Extracranial artery stenosis (ECAS) is related to individual imaging markers of cerebral small vessel disease (cSVD). However, little has been reported on the association between ECAS and the total burden of cSVD as assessed by magnetic resonance imaging (MRI). The purpose of this study was to investigate the relationship between ECAS and cSVD burden in patients with ischemic stroke of suspected small or large artery origin.

**Methods:** We reviewed consecutive patients with ischemic stroke of suspected small or large artery origin who underwent color Doppler ultrasonography and brain MRI. Bilateral extracranial cerebral arteries including common carotid artery, internal carotid artery (ICA), and proximal vertebral artery (VA, ostium, V2–3 segments) were assessed using color Doppler ultrasonography. ECAS severity was classified as no/mild stenosis, moderate stenosis, severe stenosis, or occlusion. The total cSVD score was assessed by awarding one point according to the load of each of these cSVD markers as determined using MRI; lacunar infarction, white matter hyperintensities, cerebral microbleeds, and enlarged perivascular spaces. The relationship between ECAS severity and cSVD burden according to MRI was examined.

**Results:** Two hundred and twenty one patients were included in this study (mean age 61 ± 12 years, 75.6% male). Hypertension, current smoking, hyperlipidaemia, and diabetic mellitus were frequent among the patients (67.4, 45.7, 43.9, and 36.7%, respectively), while the other vascular risk factors including previous stroke or TIA and alcohol excess were less frequent (19.0 and 15.4%, respectively). Patients with higher total cSVD burden was significantly older and had severer ECAS. The frequency of hypertension was significantly higher in patients with higher total cSVD burden. This analysis indicated that that increasing ECAS severity (from no stenosis through to 100%) was independently associated with increasing total cSVD score after adjusting for other vascular risk factors (odds ratio 1.76, 95% CI [1.16–2.69]).

**Conclusions:** In this study, high levels of ECAS from ultrasound evidence were associated with coexisting advanced cerebral cSVD in ischemic stroke patients of suspected small or large artery origin. Further studies are required to determine if and how extracranial arterial imaging helps reduce cSVD burden or improves cognitive function.

## Introduction

Cerebral small vessel disease (cSVD) related to age or hypertension is recognized as a risk factor for stroke and dementia (1). Imaging hallmarks of cSVD can be detected with magnetic resonance imaging (MRI). Lacunar, white matter hyperintensities, cerebral microbleeds, and enlarged perivascular spaces are recognized as four markers of cSVD on brain MRI (2). Recently, to capture the overall effect of cSVD on the brain more effectively, a collaborative group at Maastricht University developed a “total cSVD score” by summing all four MRI features to assess the global burden of cSVD (3). Recent studies revealed that increasing score was associated with increasing age and the presence of hypertension, male sex or smoking (4), however no other influencing factors have been fully elucidated.

Extracranial artery stenosis (ECAS), especially extracranial carotid artery stenosis, is a common disease worldwide and is one of the most important risk factors for ischemic stroke. ECAS (>50% stenosis) almost doubles the risk of ipsilateral stroke (5). Patients with either cSVD or ECAS usually share common vascular risk factors including hypertension and age (1, 6). Previous studies have indicated that asymptomatic ECAS is a risk factor not only for ischemic stroke events (7) but also for cSVD such as white matter hyperintensities and brain infarction (8) and enlarged perivascular spaces (9). The direct association of ECAS with individual cSVD markers such as white matter hyperintensities and enlarged perivascular spaces has been explored (9–11). The Northern Manhattan Study demonstrated that enlarged perivascular spaces were more frequent in those with carotid atherosclerotic plaques, and carotid plaque was a strong and consistent predictor of enlarged perivascular spaces (10). White matter hyperintensities and basal ganglia enlarged perivascular spaces were significantly greater in the unilateral hemisphere with internal carotid artery stenosis than contralateral hemisphere in another research (9). Song et al. found that the patients with aortic atheroma frequently had cSVD (11). However, little is known about the nature of the relationship between extracranial vascular stenosis and the total MRI burden of cSVD in patients with ischemic stroke. Thus, in the present study, we aimed to explore the relationship between ECAS (from no stenosis through to 100%) and combined imaging findings of cSVD in a cohort of ischemic stroke patients of suspected small or large artery origin.

## Materials and Methods

### Patient Selection

#### Inclusion Criteria

The Ethical Committee at the First Affiliated Hospital of Sun Yat-sen University reviewed and approved this study protocol. We analyzed the data of consecutive ischemic stroke patients admitted to our stroke unit from August 2016 to 2017. All enrolled patients had a definite diagnosis of ischemic stroke supported by MRI. A definite diagnosis of stroke was made on the basis of symptom duration and evidence on cerebral infarction on MRI. The symptom was defined as rapidly developed limb weakness, aphasia, ataxia, hemispatial neglect, consciousness disorder, or hemianopia, which persisted more than 24 h, with no apparent cause other than of vascular origin (12). The cerebral infarction on MRI was defined as the lesion shows hyperintensity on diffusion weighted imaging (acute infarction) or hypointensity on T1 and hyperintensity on T2 (subacute infarcts). The duration from symptom onset to admission was <30 days. We wanted to study only the strokes we thought had a high probability of being caused by intracranial arterial disease or ECAS. Patients with stroke with atherosclerotic source were included. Thus, we only recruited the ischemic stroke patients with suspected etiology which is categorized as large artery atherosclerosis or small vessel occlusion. According to TOAST classification, small artery occlusion is defined as recent subcortical infarction and <1.5 cm in diameter. Large artery atherosclerosis is defined as significant (≥50%) stenosis of the large artery relevant to the infarction.

#### Exclusion Criteria

The exclusion criteria were: (1) the stroke patients with cardioembolic stroke, stroke likely due to other demonstrated condition or undetermined classification according to the Trial of Org 10172 in Acute Stroke Treatment (TOAST) classification (13). In detail, cardioembolic stroke was defined as following: patients with arterial occlusions presumably due to an embolus arising in the heart such as atrial fibrillation on 12 lead ECG or 24 h holter during admission or on previous admissions, valvular heart disease, mobile aortic arch atheroma, evidence of intramural thrombus. Stroke likely due to other demonstrated condition was defined as following: patients with rare causes of stroke, such as hypercoagulable states, non-atherosclerotic vasculopathies, or hematologic disorders. Undetermined classified stroke was defined as following: patients have no likely etiology determined despite an extensive evaluation (CT, MRI, transcranial Doppler, electrocardiograph, 24 h holter, ultrasonography (heart echocardiogram and carotid Color Doppler ultrasonography) and laboratory examinations contain lipid, blood glucose, coagulation function test, blood rheology, immunological indicators) or have two or more potential causes of stroke so that we were unable to make a final diagnosis; (2) the patients with diseases mimicking stroke, which includes arteriovenous malformation, intracranial tumor, cerebral venous sinus thrombosis, hydrocephalus, multiple sclerosis; (3) insufficient MRI or Color Doppler ultrasonography assessment.

### Clinical Assessments and Risk Factors Definitions

At baseline, demographic data (age and sex) and history of risk factors (hypertension, diabetes mellitus, hyperlipidaemia, smoking, alcohol excess, and previous stroke) were obtained. Participants underwent an assessment of cerebrovascular risk factors during the course of the study. Hypertension was defined as systolic blood pressure ≥140 mmHg, diastolic blood pressure ≥90 mmHg, or the combination of self-reported hypertension diagnosis and use of antihypertensive medications at the time of examination (14). Diabetes mellitus was present if one of the following conditions existed: (1) fasting plasma glucose was ≥7.0 mmol/L (≥126 mg/dL), and random blood glucose was ≥200 mg/dL (11.1 mmol/L), and glycosylated hemoglobin A1–C was ≥7.0%; or (2) the person was taking oral antidiabetics drug or insulin; or (3) self-reported history of physician-diagnosed diabetes mellitus (14). Hypercholesterolemia was defied as total cholesterol ≥6.2 mmol/L, and/or the use of antidyslipidemic medication, or a self-reported history of physician-diagnosed hypercholesterolemia or self-reported of lipid-lowering medication (14). According to the National Survey on Drug Use and Health, current smoking was defined as smoking any tobacco cigarettes in the last 30 days (15). Alcohol excess was defined as more than one standard drink per day (0.355 L or 12 oz of beer, 0.118 L or 4 oz of wine, or 0.044 L or 1.5 oz of liquor or distilled spirits) (16).

### Ultrasonographic Assessment of Extracranial Cerebral Artery Stenosis

The included patients were assessed for bilateral ECAS using color Doppler ultrasonography. Symptomatic ECAS refer to sudden onset of focal neurological symptoms (on the same side of a prominent atherosclerotic lesion) matching the corresponding arterial supply, including one or more sites of ischemic stroke or transient ischemic attack. We performed Color Doppler ultrasonography on each patient within a week after admission to hospital and whenever clinically requested. We used a Philips iU22 ultrasound system equipped with a L9-3 ultrasound probe to examine the extracranial artery. Bilateral extracranial cerebral arteries including common carotid artery, extracranial internal carotid artery (ICA) and proximal vertebral artery (VA) (ostium, V2–3 segments) were assessed.

We measured the degree of stenosis as followed. First, the luminal diameter of the narrowest part of vessel was measured (*D*_stenosis_). Second, the proximal part of the vessel was taken as normal part, and the luminal diameter was measured (*D*_normal_). Third, the degree of stenosis was calculated by following equation: degree of stenosis (%) = (1–[*D*_stenosis_/*D*_normal_]) × 100%. According to (North American Symptomatic Carotid Endarterectomy, NASCET) standard (17), we classified the severity of vessel stenosis as absence of stenosis, mild stenosis (30% or less), moderate stenosis (more than 30% but < 70%), severe stenosis (at least 70% but <99%) or occlusion (100%). A mild stenosis is defined as a visible wall irregularity impinging on the vascular lumen, without blood flow abnormality on Doppler, the change peak systolic frequency <2.0 kHz. A moderate stenosis (more than 30% but <69%) is defined as a visible wall abnormality with luminal narrowing up to 69% diameter and the change peak systolic frequency <4 kHz or a peak systolic velocity of <120 cm/s or a peak systolic velocity ratio of 2.4. A severe stenosis (at least 70% but <99%) is defined as a visible abnormality with a systolic frequency change of >4 kHz or a peak systolic velocity of >120 cm/s. Occlusion (100%) is defined as no blood flow signal (18). Two experienced neurologists independently reviewed all images and were blinded to clinical data and Color Doppler ultrasonography findings.

### Evaluation of Total cSVD Burdens

All four MRI markers of cSVD (lacunar, white matter hyperintensities, cerebral microbleeds and enlarged perivascular spaces) were incorporated to represent the total burden of cSVD using methods from previous studies (2–4, 19).

#### Lacunar

Lacunar is defined as a round or ovoid, subcortical, fluid-filled cavity, consistent with a previous small subcortical infarct or hemorrhage in the territory of one perforating arteriole (2). The lacunar infarct is typically hypointensive on T2-weighted imaging and fluid-attenuated inversion recovery imaging but hypointensive on T1-weighted imaging with a diameter 3–15 mm (2). One point was given if at least one lacunar infarction located in the internal or external capsule, thalamus, basal ganglia or brain stem was present in either cerebral hemisphere on MRI and not compatible with the incident stroke (3). We did not consider that the Lacunar is <20 mm as in the previous literature but 3–15 according to the current literature (2).

#### White Matter Hyperintensities

White matter hyperintensities is defined as signal abnormality of variable size in the white matter. It shows the following characteristics: it is hyperintense on T2-weighted sequences and hyperintensity on T2-weighted images such as fluid-attenuated inversion recovery, isointense, or hypointense on T1-weighted sequences (2). Fazekas score was used to evaluate the severity of white matter hyperintensities as followed: Periventricular hyperintensity was graded as 0 = absence, 1 = “caps,” or pencil-thin lining, 2 = smooth “halo,” 3 = irregular periventricular hyperintensities extending into the deep white matter. Separate deep white matter hyperintense signals were rated as 0 = absent, 1 = punctate foci, 2 = beginning confluence of foci, 3 = large confluent areas (20, 21). One point was given for if Fazekas score is ≥2 in deep white matter or >2 in periventricular white matter (3).

#### Cerebral Microbleeds

Damage of small vessel wall would cause disrupted brain blood barrier. The component of blood such as hemoglobin would leak in the brain through the damaged small vessel wall, which is the pathological nature of cerebral microbleed (2). When hemoglobin is degraded, hemosiderin, the degradation product, deposits in the brain. The hemosiderin can cause significant signal loss on MRI, especially on susceptibility-weighted imaging. Thus, cerebral microbleeds is defined as small and hypointense lesions that are visible on susceptibility-weighted imaging with a diameter <10 mm (2). One point was given if evidence of at least one cerebral microbleeds was present in the internal or external capsule, basal ganglia or thalamus in either cerebral hemisphere (3). We did not count cerebral microbleeds in the cortex is thought to be associated with amyloidosis rather than atherosclerosis.

#### Enlarged Perivascular Spaces

Enlarged perivascular spaces are fluid-filled spaces that follow the typical course of a vessel as it goes through gray or white matter. The spaces have signal intensity similar to that of CSF on all sequences. They appear linear, round or ovoid with a diameter generally smaller than 3 mm (2). Enlarged perivascular spaces on the slide with the highest number in 1 hemisphere was counted. We graded them with a formerly used 3-category ordinal scale (0–10; 10–25; >25). If there were more than 10 enlarged perivascular spaces on the hemisphere with the highest number, one point was scored in the analysis of total cSVD burden (3).

#### Calculation of Total cSVD Score

The total cSVD score is used to represent the total MRI burden of cSVD (3, 4, 19). The total cSVD score was calculated by summing up the score of the above cSVD markers for each patient (3, 4, 19). Therefore, the range of total cSVD scores was 0–4 points. Furthermore, the patients were assigned to moderate total cSVD burden group if their total cSVD score was 1 or 2. If their total cSVD score was 3 or 4, the patients were assigned to severe total cSVD burden group. If their total cSVD score was 0, the patients were assigned to absent/mild total cSVD burden group. Therefore, patients were assigned to absent/mild (total cSVD score 0), moderate (total cSVD score 1 and 2), or severe (total cSVD score 3 and 4) total cSVD burden group.

### Statistical Analysis

Data were presented as the percentage (number) or frequency for discrete variables and as the median for continuous variables. The chi-squared test, ANOVA and the Kruskal-Wallis test were used to detect differences where appropriate, and the Bonferroni method was used for *post hoc* analysis. Ordinal regression was used to assess the ordinal correlation between the severity of ECAS (mild [0–30%], moderate [31–69%], severe [71–99%], and occlusion [100%]) and total cSVD burden, in which absent/mild, moderate or severe total cSVD burden was assigned as 0, 1, or 2, respectively. Age, sex, or vascular risk factors (hypertension, diabetes mellitus, hyperlipidemia, smoking, alcohol excess, and previous stroke) were ‘adjusted. The odds ratio (OR) and 95% confidence interval (CI) were obtained. Kappa**-**value was used to evaluate the inter-rater reliability of total MRI burden analysis between two neurologists. When the *P* < 0.05, the results were considered statistically significant. All statistical analyses were performed with the software IBM SPSS Statistics for Windows (Version 22.0, IBM Corp., Armonk, New York, USA).

## Results

### Patient Characteristics and Clinical Variations

Two hundred and twenty-one patients were included in the study. Among all patients, small artery occlusion stroke (according to TOAST classification, small artery occlusion is defined as recent subcortical infarction and <1.5 cm in diameter) made up for 57.3% of all stroke subtype. Large-artery atherosclerosis stroke (according to TOAST classification, large artery atherosclerosis is defined as significant (≥50%) stenosis of the large artery relevant to the infarction) made up for 42.7% of all stroke subtype. The demographic and clinical characteristics of all the included patients are summarized in Table 1. The median age of the participants was 61 ± 12 years, and 75.6% of them were male. Vascular risk factors such as hypertension, current smoking, hyperlipidemia, and diabetic mellitus were frequent among the all patients (67.4, 45.7, 43.9, and 36.7%, respectively), while there were fewer patients with other vascular risk factors including previous stroke/TIA and alcohol excess (19.0 and 15.4%, respectively).

**Table 1:** Clinical characteristics of included patients.

		**Total cSVD Burden**		
	**All (*n* = 221)**	**Absent/Mild (*n* = 32)**	**Moderate (*n* = 105)**	**Severe (*n* = 84)**
Age (years)^*^	61 ± 12	49 ± 10	60 ± 10	67± 11
Male, *n* (%)	167 (75.6)	25 (78.1)	82 (78.1)	60 (71.4)
Hypertension, *n* (%)^*^	149 (67.4)	16 (50.0)	67 (63.8)	66 (78.6)
Diabetes mellitus, *n* (%)	81 (36.7)	11 (34.4)	40 (38.1)	30 (35.7)
Hyperlipidemia, *n* (%)	97 (43.9)	18 (56.3)	47 (44.8)	32 (38.1)
History of stroke/TIA, *n* (%)	42 (19.0)	3 (9.4)	19 (18.1)	20 (23.8)
Alcohol excess, *n* (%)	34 (15.4)	5 (15.6)	18 (17.1)	11 (13.1)
Current smoking, *n* (%)	101 (45.7)	17 (53.1)	54 (51.4)	30 (35.7)
Previous coronary artery disease	4 (1.8)	0 (0)	2 (1.9)	2 (2.4)
**EXTRACRANIAL ARTERY STENOSIS**, ***n*** (%)^*^				
Absent/mild (0–30%)	195 (88.2)	30 (93.8)	99 (94.3)	66 (78.6)
Moderate (31–69%)	8 (3.6)	1 (2.7%)	1 (1.0)	6 (7.1)
Severe (70–99%)	8 (3.6)	0	2 (1.9)	6 (7.1)
Occlusion (100%)	10 (4.5)	1 (2.7%)	3 (2.9)	6 (7.1)

*cSVD, cerebral small vessel disease; TIA, transient ischemic attack*.

* *P < 0.05 compared with Absent group*.

The proportions of patients with absent or mild, moderate, and severe total cSVD burden were 15.5, 47.5, and 38.0%, respectively. The inter rater reliability of the scoring is high with κ values 0.83 for total cSVD burden analysis. Among 221 patients, extracranial carotid stenosis was found in 217 patients, and extracranial VA stenosis was found in only 4 patients (see Table 2). The numbers of patients with absent or mild, moderate, severe, and occlusion ECAS were 195, 8, 9, and 9, respectively. Among patients with extracranial VA stenosis, there were 3 cases with mild stenosis and 1 with severe stenosis. In total, there were 57 patients with symptomatic ECAS. Three patients had symptomatic VA stenosis. The remaining 54 patients had symptomatic carotid stenosis. Among the patients with symptomatic common carotid stenosis (*n* = 15), there were 11, 1, 2, and 1 patients found with mild, moderate, severe and occlusion of common carotid artery, respectively. Among the patients with asymptomatic common carotid stenosis (*n* = 71), there were 67, 1, 0, and 3 patients found with mild, moderate, severe, and occlusion of common carotid artery, respectively. Among the patients with symptomatic extracranial internal carotid artery stenosis (*n* = 39), there were 29, 4, 5, and 1 patients found with mild, moderate, severe, and occlusion of extracranial internal carotid artery, respectively. Among the patients with asymptomatic extracranial internal carotid artery stenosis (*n* = 92), there were 85, 2, 1, and 4 patients found with mild, moderate, severe and occlusion of extracranial internal carotid artery, respectively. Among the patients with symptomatic extracranial VA stenosis (*n* = 3), there were 2 and 1 patients found with mild and severe of extracranial VA, respectively. Among the patients with asymptomatic extracranial VA stenosis (*n* = 1), there was 1 patients found with mild of extracranial VA. A comparison of clinical characteristics among patients with different degrees of total cSVD load showed a significant difference in age among patients with different total cSVD scores. Specifically, patients with higher cSVD scores were older (*P* < 0.05). Additionally, the frequency of hypertension and the severity of ECAS were different among patients with different total cSVD scores (*P* < 0.05). Increasing blood pressure, severer ECAS, there were more common with increasing cSVD score (*P* < 0.05). However, these groups did not significantly differ in terms of other clinical characteristics.

**Table 2:** Degree of stenosis of carotid artery and vertebral artery.

	**CCA (*****n*** **=** **86)**		**ICA (*****n*** **=** **131)**		**VA (*****n*** **=** **4)**	
	**Symptomatic** **(*n* = 15)**	**Asymptomatic** **(*n* = 71)**	**Symptomatic** **(*n* = 39)**	**Asymptomatic** **(*n* = 92)**	**Symptomatic** **(*n* = 3)**	**Asymptomatic** **(*n* = 1)**
Absent/mild (0–30%, *n* = 195)	11	67	29	85	2	1
Moderate (31–69%, *n* = 8)	1	1	4	2	0	0
Severe (70–99%, *n* = 9)	2	0	5	1	1	0
Occlusion (100%, *n* = 8)	1	3	1	4	0	0

*CCA, common carotid artery; ICA, extracranial internal carotid artery; VA, vertebral artery*.

### Associations Between Extracranial Vascular Stenosis and Total MRI Burden of cSVD

We performed ordinal regression after multivariate adjustment for possible confounders includes age, sex, and vascular risk factors (hypertension, diabetes mellitus, hyperlipidaemia, smoking, alcohol excess, and previous stroke). This analysis indicated that that increasing ECAS severity (from no stenosis through to 100%) was independently associated with increasing total cSVD score after adjusting for other vascular risk factors (odds ratio 1.76, 95%CI [1.16–2.69]) (Table 3).

**Table 3:** Correlation between total cSVD score and the severity of extracranial artery stenosis in multivariable ordinal regression analysis.

	**Unadjusted OR (95% CI)**	**Age-and sex-adjusted OR (95% CI)**	**Multivariate adjusted OR (95% CI)**	***P-value***
Extracranial carotid artery stenosis	1.71 (1.16–2.53)	1.73 (1.15–2.62)	1.76 (1.16–2.69)	*P* < 0.05

*CI, confidence interval; OR, odds ratio. ECAS, Extracranial carotid artery stenosis. Multivariate adjusted: age, sex, or vascular risk factors (hypertension, diabetes mellitus, hyperlipidaemia, smoking, alcohol excess, and previous stroke) were adjusted*.

## Discussion

The total cSVD score is an overall score for evaluating the burden of cSVD on MRI (3). The score provides an overview on cSVD rather than information about individual cSVD features (4). Furthermore, total cSVD score is simple to implement as long as a standardized definition of each feature is adopted (4). This scoring system could be useful for rapid stratification of cSVD patients in clinical trials (4).

Recent studies reveal that total cSVD burden correlates with blood pressure, cognitive performance, and stroke outcome. Arba et al. suggest that the total cSVD burden is negatively associated with stroke outcomes after intravenous thrombolysis (22). The higher total cSVD burden is associated with decreased performance on tests of information processing speed (19) and lower general cognitive ability (23). Klarenbeek et al. found a positive association of ambulatory blood pressure levels with total cSVD burden in patients with first-ever lacunar stroke (3). Recent studies have revealed that total cSVD burden was associated with age and hypertension (4). Similarly, our study indicated that age and hypertension independently associated with total cSVD burden after adjusting for the other vascular risk factors.

ECAS, as branches of the aorta, are known to be involved in microemboli (24) or the reduction of cerebral blood flow (25). Previous studies have indicated that asymptomatic ECAS is a risk factor for ischemic stroke events (7) as well as for cSVD (9). Wang et al. found a positive correlation between asymptomatic ECAS and the occurrence of new cerebrovascular and cardiovascular disease, especially brain infarction events. Increased carotid intima-media thickness and carotid plaque are relevant to the occurrence of vascular events (7). ECAS is also a risk factor for cognitive decline (26). However, the underlying mechanisms of ECAS relating cognitive decline is not clear. Hemodynamic dysfunction and microemboli arising from atherosclerotic plague from extracranial cerebral artery may contribute to the development of cSVD and may further correlate with cognitive decline. Our study showed positive correlation with ECAS and total cSVD burden and may support the above mentioned hypothesis. Our findings did not show a causation association between ECAS and cSVD. They are likely to be caused by the same risk factors, but that ECAS may make cSVD worse via reduced flow and embolization. The basic principle for the treatment of carotid stenosis is to improve blood supply to the brain and prevent ischemic stroke and TIA. At present, the main clinical treatments include medical intervention (encouraging healthy life style habits and the best medical treatment), carotid endarterectomy and carotid artery stenting (CAS) (27). Thus, treating ECAS may help to slow cognition decline. It is unproven as to whether or not CEA or CAS can improve cognition. So far the studies show inconsistent results because of using heterogeneous of standardization of neuropsychological testing, follow-up timing and incorporation of neuroimaging, thus do not allow for accurate conclusions to be drawn (28).

Furthermore, a systematic review of management guidelines explains how there is no randomized trial evidence of benefit from carotid endarterectomy compared to current medical treatment alone, how asymptomatic persons are less likely to benefit from a carotid procedure than symptomatic persons and how stenting is associated with higher stroke and death rates than endarterectomy (29). Stroke and cognitive dysfunction prevention without serious complications is the main goal of successful treatment. Thus, risk-benefit assessment should be implemented before CAS or CEA (30). The prevalence of vascular risk factors (such as hypertension, diabetes mellitus, smoking, and so on) was high in our study. Recent research showed that the Mediterranean diet, in moderate to vigorous intensity activity (lasting 40 min on average, three to four times per week) and smoking cessation reduce the risk of ECAS (31). Pharmacologic treatment including antihypertensives, statins, and antiplatelet agents can stabilize atherosclerotic lesions reduce the risk and progression of ECAS (32).

Color Doppler ultrasonography is a convenient and safe method that can be used to study the ECAS (from no stenosis through to 100%) (33). Our data revealed that the increasing severity of ECAS (from no stenosis through to 100%) as measured by color Doppler ultrasonography was an independently predictor of the heavier total cSVD burden after adjusted by age, sex, or vascular risk factors (hypertension, diabetes mellitus, hyperlipidaemia, smoking, alcohol excess, and previous stroke), which consistent with previous results that ECAS and cSVD are directly related (9–11). Similar to previous research (7), a few of patients (only 4 among the 221) had extracranial VA stenosis, the remaining patients had extracranial carotid arterial stenosis. Hence, our study results reveal that ECAS (from no stenosis through to 100%), particularly in the proximal ICA, was a risk factor for the total burden of cSVD. The correlation of ECAS with MRI signs of cSVD indicates that ultrasound imaging of the extracranial cerebral arteries may be the more convenient and cheaper way to quantify cerebral arterial disease in future studies. Assessing the severity of ECAS may be a convenient surrogate method for evaluating the development of total cSVD burden, as well as for follow-up for subclinical signs of disease progression, particularly in remote rural areas or hospitals without magnetic resonance equipment. However, such clinical potential requires testing in further studies.

Some limitations of this observational study should be considered. First, some patients with more severe ischemic stroke were unable to complete MRI or Color Doppler ultrasonography examinations, severe ECAS and severe total burden of cSVD were rarely found in our sample, which introduces potential selection bias. Furthermore, we defined ischemic stroke according to the clinical symptoms and positive imaging findings. DWI abnormalities are absent in up to 30% of ischemic strokes particularly when the lesions involve small volumes of brain tissue and are in the posterior circulation and in patients who received imaging very soon or relatively late after the clinical features of stroke onset (12). Excluding these patients without MRI abnormalities is the other potential bias of this study. Second, Color Doppler ultrasonography to evaluate ECAS was performed by several different operators in our study. Duplex ultrasound is now a widely-used method of assessing the degree of stenosis, the accuracy of the measurements may vary from one operator to the next. Furthermore, ECAS was not evaluated with multiple imaging methods in our study. Thus, the ultrasonographic data cannot compared with other imaging form like computed tomography angiography, magnetic resonance angiography or digital subtraction angiography. The Duplex ultrasonography may overestimate the degree of stenosis (34). However, any disagreement about the results was resolved by specialist team. Thus, the variability was minimized. Third, in our study, we aimed to investigate the ischemic stroke of atherosclerotic etiology within the range from intracranial artery to extracranial artery distal to aorta. Thus, we excluded the cardiac embolic stroke, stroke of other demonstrated etiology and undetermined classified stroke. Last, it is a limitation of our study that we do not have information about the nature of the non-invasive arterial disease treatments (involving lifestyle habits and medications) received by our patients. These treatments were likely to have had an impact on the brain and arterial imaging results of our study.

## Conclusion

In summary, we demonstrated that increasing ECAS from 0 through to 100% was positively correlated with increasing evidence of cSVD as determined using MRI in patients with ischemic stroke of suspected small or large arterial origin. Assessing the level of ECAS may be a convenient method for evaluating the severity of total cSVD MRI burden in patients with ischemic stroke of suspected small or large artery origin. It is easier or cheaper to use US rather than MRI as a screening tool or as surrogate target for arterial disease treatment. Further studies are required to determine if extracranial arterial imaging helps reduce cSVD burden and stroke risk or improves cognition, particularly in the context of current best methods of reducing arterial disease risk by only using lifestyle modification and medication.

## Ethics Statement

This study was carried out in accordance with the recommendations of Guideline of Clinical Research, The Ethical Committee at the First Affiliated Hospital of Sun Yat-sen University with written informed consent from all subjects. All subjects were informed that their clinical data would be recorded in clinical database and be reviewed for research purpose. All subjects gave written informed consent in accordance with the Declaration of Helsinki. The protocol was approved by The Ethical Committee at the First Affiliated Hospital of Sun Yat-sen University.

## Author Contributions

TL, JL, and JZ designed the study. TL obtained the data for the work. JL analyzed the data. TL and JL interpreted the data and drafted the study. NW, LP, HY, and BW provided advice for designing and drafting the work. JZ revised all data and critically revised the paper for important intellectual content. All authors approved the final version of the paper to be published.

### Conflict of Interest Statement

The authors declare that the research was conducted in the absence of any commercial or financial relationships that could be construed as a potential conflict of interest.

## References

[1] Pantoni L. Cerebral small vessel disease: from pathogenesis and clinical characteristics to therapeutic challenges. Lancet Neurol. (2010) 9:689–701. 10.1016/S1474-4422(10)70104-6 20610345

[2] Wardlaw JM Smith C Dichgans M. Mechanisms of sporadic cerebral small vessel disease: insights from neuroimaging. Lancet Neurol. (2013) 12:483–97. 10.1016/S1474-4422(13)70060-7 23602162 PMC3836247

[3] Klarenbeek P van Oostenbrugge RJ Rouhl RP Knottnerus IL Staals J. Ambulatory blood pressure in patients with lacunar stroke: association with total MRI burden of cerebral small vessel disease. Stroke. (2013) 44:2995–9. 10.1161/STROKEAHA.113.002545 23982717

[4] Staals J Makin SD Doubal FN Dennis MS Wardlaw JM. Stroke subtype, vascular risk factors, and total MRI brain small-vessel disease burden. Neurology. (2014) 83:1228–34. 10.1212/WNL.0000000000000837 25165388 PMC4180484

[5] Abbott AL Bladin CF Levi CR Chambers BR. What should we do with asymptomatic carotid stenosis? Int J Stroke. (2007) 2:27–39. 10.1111/j.1747-4949.2007.00096.x 18705984

[6] Chuang ML Gona P Oyama-Manabe N Manders ES Salton CJ Hoffmann U. Risk factor differences in calcified and noncalcified aortic plaque: the Framingham Heart Study. Arterioscler Thromb Vasc Biol. (2014) 34:1580–6. 10.1161/ATVBAHA.114.303600 24833796 PMC4099001

[7] Wang D Wang J Jin C Ji R Wang A Li X. Asymptomatic extracranial artery stenosis and the risk of cardiovascular and cerebrovascular diseases. Sci Rep. (2016) 6:33960. 10.1038/srep33960 27650877 PMC5030632

[8] Moroni F Ammirati E Magnoni M D'Ascenzo F Anselmino M Anzalone N. Carotid atherosclerosis, silent ischemic brain damage and brain atrophy: a systematic review and meta-analysis. Int J Cardiol. (2016) 223:681–7. 10.1016/j.ijcard.2016.08.234 27568989

[9] Sahin N Solak A Genc B Akpinar MB. Dilatation of the Virchow-Robin spaces as an indicator of unilateral carotid artery stenosis: correlation with white matter lesions. Acta Radiol. (2015) 56:852–9. 10.1177/0284185114544243 25140058

[10] Gutierrez J Rundek T Ekind MS Sacco RL Wright CB. Perivascular spaces are associated with atherosclerosis: an insight from the Northern Manhattan Study. Am J Neuroradiol. (2013) 34:1711–16. 10.3174/ajnr.A3498 23557952 PMC4380264

[11] Song TJ Kim YD Yoo J Kim J Chang HJ Hong GR. Association between aortic atheroma and cerebral small vessel disease in patients with ischemic stroke. J Stroke. (2016) 18:312–20. 10.5853/jos.2016.00171 27488980 PMC5066433

[12] Abbott AL Silvestrini M Topakian R Golledge J Brunser AM de Borst GJ. Optimizing the definitions of stroke, transient ischemic attack, and infarction for research and application in clinical practice. Front Neurol. (2017) 8:537. 10.3389/fneur.2017.00537 29104559 PMC5654955

[13] Adams HP Bendixen BH Kappelle LJ Biller J Love BB Gordon DL. Classification of subtype of acute ischemic stroke. Definitions for use in a multicenter clinical trial. TOAST. Trial of Org 10172 in Acute Stroke Treatment. Stroke. (1993) 24:35–41. 7678184 10.1161/01.str.24.1.35

[14] Ong YT De Silva DA Cheung CY Chang HM Chen CP Wong MC. Microvascular structure and network in the retina of patients with ischemic stroke. Stroke. (2013) 44:2121–7. 10.1161/STROKEAHA.113.001741 23715958

[15] Ryan H Trosclair A Gfroerer J. Adult current smoking: differences in definitions and prevalence estimates–NHIS and NSDUH, 2008. J Environ Public Health. (2012) 2012:918368. 10.1155/2012/918368 22649464 PMC3357540

[16] Adams MK Chong EW Williamson E Aung KZ Makeyeva GA Giles GG. 20/20–Alcohol and age-related macular degeneration: the Melbourne Collaborative Cohort Study. Am J Epidemiol. (2012) 176:289–98. 10.1093/aje/kws004 22847604

[17] North American Symptomatic Carotid Endarterectomy Trial Collaborators Barnett HJM Taylor DW Haynes RB Sackett DL Peerless SJ. Beneficial effect of carotid endarterectomy in symptomatic patients with high-grade carotid stenosis. N Engl J Med. (1991) 325:445–53. 1852179 10.1056/NEJM199108153250701

[18] Bel'skaia GN Luk'ianchikova LV. [Quality of life of patients with ischemic stroke in the vertebrobasilar system]. Zh Nevrol Psikhiatr Im S S Korsakova. (2013) 113 (Pt. 2):24–8. 24430051

[19] Huijts M Duits A van Oostenbrugge RJ Kroon AA de Leeuw PW Staals J. Accumulation of MRI markers of cerebral small vessel disease is associated with decreased cognitive function. A Study in First-Ever Lacunar Stroke and Hypertensive Patients. Front Aging Neurosci. (2013) 5:72. 10.3389/fnagi.2013.00072 24223555 PMC3818574

[20] Fazekas F Kleinert R Offenbacher H Schmidt R Kleinert G Payer F. Pathologic correlates of incidental MRI white matter signal hyperintensities. Neurology. (1993) 43:1683–9. 8414012 10.1212/wnl.43.9.1683

[21] Fazekas F Chawluk JB Alavi A Hurtig HI Zimmerman RA. MR signal abnormalities at 1.5 T in Alzheimer's dementia and normal aging. Am J Roentgenol. (1987) 149:351–6. 3496763 10.2214/ajr.149.2.351

[22] Arba F Inzitari D Ali M Warach SJ Luby M Lees KR. Small vessel disease and clinical outcomes after IV rt-PA treatment. Acta Neurol Scand. (2017) 136:72–7. 10.1111/ane.12745 28233290

[23] Staals J Booth T Morris Z Bastin ME Gow AJ Corley J. Total MRI load of cerebral small vessel disease and cognitive ability in older people. Neurobiol Aging. (2015) 36:2806–11. 10.1016/j.neurobiolaging.2015.06.024 26189091 PMC4706154

[24] Madani A Beletsky V Tamayo A Munoz C Spence JD. High-risk asymptomatic carotid stenosis: ulceration on 3D ultrasound vs. TCD microemboli. Neurology. (2011) 77:744–50. 10.1212/WNL.0b013e31822b0090 21849642

[25] Claus JJ Breteler MM Hasan D Krenning EP Bots ML Grobbee DE. Vascular risk factors, atherosclerosis, cerebral white matter lesions and cerebral perfusion in a population-based study. Eur J Nucl Med. (1996) 23:675–82. 8662102 10.1007/BF00834530

[26] Man BL Fu YP Wong A Chan YY Lam W Hui AC. Cognitive and functional impairments in ischemic stroke patients with concurrent small vessel and large artery disease. Clin Neurol Neurosurg. (2011) 113:612–16. 10.1016/j.clineuro.2011.04.001 21530070

[27] Hatano T Tsukahara T Miyakoshi A Arai D Yamaguchi S Murakami M. Stent placement for atherosclerotic stenosis of the vertebral artery ostium: angiographic and clinical outcomes in 117 consecutive patients. Neurosurgery. (2011) 68:108–16. 10.1227/NEU.0b013e3181fc62aa 21099720

[28] Paraskevas KI Lazaridis C Andrews CM Veith FJ Giannoukas AD. Comparison of cognitive function after carotid artery stenting versus carotid endarterectomy. Eur J Vasc Endovasc Surg. (2014) 47:221–31. 10.1016/j.ejvs.2013.11.006 24393665

[29] Abbott AL Paraskevas KI Kakkos SK Golledge J Eckstein HH Diaz-Sandoval LJ. Systematic review of guidelines for the management of asymptomatic and symptomatic carotid stenosis. Stroke. (2015) 46:3288–301. 10.1161/STROKEAHA.115.003390 26451020

[30] Noiphithak R Liengudom A. Recent update on carotid endarterectomy versus carotid artery stenting. Cerebrovasc Dis. (2017) 43:68–75. 10.1159/000453282 27898402

[31] Wabnitz AM Turan TN Symptomatic carotid artery stenosis: surgery, stenting, or medical therapy? Curr Treat Options Cardiovasc Med. (2017) 19:62 10.1007/s11936-017-0564-0 28677035 PMC5496976

[32] Migrino RQ Bowers M Harmann L Prost R LaDisa JF. Carotid plaque regression following 6-month statin therapy assessed by 3T cardiovascular magnetic resonance: comparison with ultrasound intima media thickness. J Cardiovasc Magn Reson. (2011) 13:37. 10.1186/1532-429X-13-37 21812992 PMC3166901

[33] Vucaj-Cirilović V Lucić M Petrović K Nikolić O Govorcin M Stojanović S. Color Doppler ultrasonography and multislice computer tomography angiography in carotid plaque detection and characterization. Vojnosanit Pregl. (2011) 68:423–9. 10.2298/VSP1105423V 21739910

[34] Netuka D Belšán T Broulíková K Mandys V Charvát F Malík J. Detection of carotid artery stenosis using histological specimens: a comparison of CT angiography, magnetic resonance angiography, digital subtraction angiography and Doppler ultrasonography. Acta Neurochir. (2016) 158:1505–14. 10.1007/s00701-016-2842-0 27255656

